# Score for Differentiating Pleural Tuberculosis from Malignant Effusion

**DOI:** 10.3390/medsci7030036

**Published:** 2019-02-26

**Authors:** Alejandra González, Mariano Fielli, Adrián Ceccato, Carlos Luna

**Affiliations:** 1 Pneumonology, Hospital Nacional Profesor Alejandro Posadas, Haedo, El Palomar, Buenos Aires 1684, Argentina; mhfielli@hotmail.com (M.F.); aceccato@clinic.cat (A.C.); 2 Pneumonology, Hospital de Clínicas, Buenos Aires 1120, Argentina; dr.cm.luna@gmail.com

**Keywords:** tuberculosis, neoplasia, pleural effusion, score

## Abstract

Differential diagnosis of lymphocytic pleural effusions between tuberculous (TBE) and malignant (ME) effusion is usually difficult in daily practice. Our aim was to develop a score to differentiate TBE from ME effusions. A cohort of 138 consecutive patients with pleural effusion was prospectively studied from May 2014 through June 2017. Glucose, lactate dehydrogenase (LDH), proteins, white cell count, lactic acid, and pH in the pleural fluid were measured. Pleural effusions other than lymphocytic, patients with a final diagnosis other than tuberculosis or malignancy, and patients who met Light’s criteria for transudate were excluded. Eighty-two samples (47 TBE and 35 ME) were included in the analysis. Using logistic regression analysis and Wald test, we developed a score including age, glucose, proteins, and lactic acid. The receiver operating characteristic curve (ROC) for the score was determined, and the area under the curve (AUC) was calculated. A cutoff of eight points was required to achieve 93.5% sensitivity, 78% specificity, and a likelihood ratio of 4.26 to distinguish tuberculosis from malignant pleural effusion. The AUC of the score was 0.915 (95% CI = 0.82–0.96).

## 1. Introduction

The differential diagnosis of pleural effusions with lymphocyte-predominant exudate requires careful exploration. The parameters that are usually available and commonly measured in pleural effusion are useful for the separation of transudates from exudates but often they do not contribute to the diagnosis of the underlying disease [1]. Also, the procedures that leads to a definitive diagnosis (i.e., biopsy and/or culture) could result in a delay that threats the adequate management of the disease and its outcome. In daily practice, it is usually difficult to differentiate tuberculous (TBE) from malignant effusion (ME) in the absence of other signs or radiological manifestations of the disease. TBE can occur in the absence of other radiological evidence of pulmonary tuberculosis and may be due to primary or extra-primary disease, depending on the population studied [2]. Tuberculous pleurisy is the most common form of extra-pulmonary tuberculosis. 

The presence of malignant cells in pleural effusion or pleura tissue is observed in disseminated or advanced neoplastic disease and leads to poor outcomes, highlighting its importance as a prognostic factor [3]. Currently, lung and breast cancer are the most common metastatic tumors to the pleura in men and women, respectively. Together, both malignancies account for about 70% of all malignant pleural effusions [4,5].

The aim of this study was to develop a score to differentiate tuberculous pleurisy from malignant effusion using widely available variables. 

## 2. Materials and Methods

All the patients with a diagnosis of pleural effusion were assessed for a three-year period (from May 2014 through June 2017). Only exudative lymphocytic pleural effusions were included. The patients with Human Immunodeficiency Virus (HIV) infection were excluded. The diagnosis of ME was defined as the presence of atypical cells in pleural fluid or by the histopathological examination of pleural tissue. The diagnosis of TBE was defined as a positive culture of pleural fluid or tissue or the presence of caseum granulomas in the pleural biopsy. Glucose, lactate dehydrogenase (LDH), proteins, white cell count, pH, and lactic acid were measured in pleural fluid. For the determination of lactic acid, a simple and fast enzymatic method employing the reactive commercial Kit-Roche was used.

The R Project for Statistical Computing (version 2.15.3) software was used for the statistical analysis. Median (interquartile range (IQR)) was used for continuous variables, with non-normal distribution and mean (standard deviation (SD)) for those with normal distribution. Categorical variables were compared using the Chi-square test or the Fisher exact test. Continuous variables were compared using the *t*-test or the nonparametric Mann–Whitney test. Logistic regression analysis was performed, and a set of variables with the highest Wald statistic test value were used to develop the score. These variables were categorized into three groups, and each one was rated from one to three points according to the likelihood of tuberculosis disease (the higher score predicts a higher probability of tuberculosis). The receiver Operating Characteristic Curve (ROC) was generated for the score, and the AUC was calculated. The significance level was set to *p* < 0.05.

The study was conducted in accordance with the Declaration of Helsinki 2013, Law 25.326 of the Ministry of Health, and the protocol was approved by the ethics committee of Dr. Vicente Federico Del Giùdice (ref: 001LUPOSOAD/10).

## 3. Results

**Figure 1:**
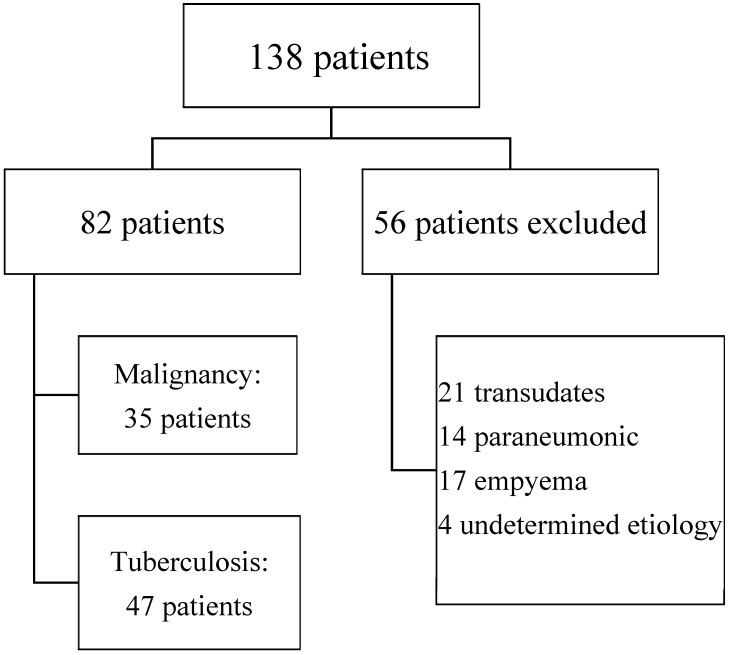
Flowchart of patient inclusion.

We included 138 patients with pleural effusion. Fifty-six patients were excluded, presenting transudate (21), parapneumonic effusion (14), and empyema (17). Four patients were followed up for up to 12 months without a confirmed etiology. Eighty-two samples of exudative lymphocytic pleural effusion were included in the analysis. Cytological or histological confirmation of neoplasia was obtained in 35 (43%) of them, and 47 (57%) were diagnosed as TBE (Figure 1).

Patients with TBE diagnosis were younger (mean in years: 37.6 ± 18.0 versus 61.9 ± 14.0, *p* < 0.001), and no differences in gender were observed (*p* = 0.62). There were statistically significant differences in lactic acid (4.9 IQR 3.52–6.0 versus 2.4 IQR 1.82–3.72, *p* < 0.001), glucose (0.7 IQR 0.6–0.81 versus 1.04 IQR 0.92–1.35, *p* < 0.001), proteins (5.1 IQR 4.5–5.7 versus 3.95 IQR 2.3–4.8, *p* < 0.001), LDH (868 IQR 533–1289 versus 355 IQR 272–585, *p* < 0.001), pH (7.30 IQR 7.30–7.40 versus 7.40 IQR 7.35–7.40, *p* < 0.004), and white cell count (1600 IQR 897–3067 versus 650 IQR 350–1770, *p* < 0.005) for TBE and ME effusions, respectively (Table 1).

In the multivariate analysis, only glucose remained statistically different. The first four variables with the highest Wald test value were included in the score: lactic acid, glucose, age, and proteins. Each one was categorized in three groups (Table 2).

The selected continuous variables were divided into three categories, and a score of one to three points was assigned to each one.

**Figure 2:**
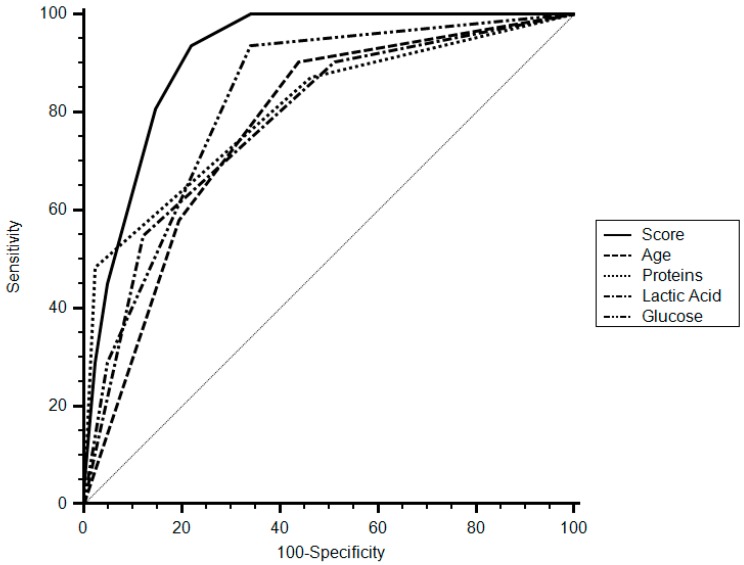
Receiver operating characteristic curve (ROC) for score and categorized variables. AUC, area under the curve.

A cut-off of eight points of the score had a specificity of 78% (95% CI 62.4–89.4), sensitivity of 93.5% (95% CI 78.6–99.2), and a positive likelihood ratio (LR) of 4.26 (95% CI 3.5–5.1) for the diagnosis of tuberculous pleurisy. The area under curve (AUC) of the score was 0.915 (95% CI 0.825–0.968, *p* < 0.001) (Figure 2).

## 4. Discussion

In this study, we analyzed a group of patients with lymphocytic pleural effusion and developed a score using widely available variables to determine the diagnosis of TBE. The score had a high sensitivity and specificity for the diagnosis of tuberculous effusion. The AUC of the score was 0.915, which surpasses the threshold level of 0.75 that was reported as clinically useful.

We decided to use the level of lactic acid in pleural fluid because it is easier to categorize than the pH and is widely available. Also, the measurement of lactic acid in cerebrospinal, pleural, and ascitic fluid has been already used to differentiate bacterial infectious etiologies from others [6,7]. Gastrin et al. studied 198 patients and found that empyema had the highest level of lactic acid, whereas malignancy had the lowest [8].

The common values used to differentiate pleural effusions, such as glucose level, are often decreased in tuberculosis [2]. We found significant differences between the two groups, although the values were close to the physiological levels. A pleural-fluid protein level greater than 5 g/dL suggests tuberculous pleuritis [9]. We obtained a median protein level of 5.1 g/dL in the tuberculosis group.

In our study, patients with pleural tuberculosis were younger. However, this could vary according to the study population and the prevalence of the disease. The incidence of tuberculosis in Argentina is 26.5/100,000/year. In developed countries, the average age tends to be higher, and reactivation is the cause in the majority of cases [2].

The bacteriological confirmation of tuberculous pleuritis is usually difficult. In most series of immunocompetent patients, less than 40% of the cultures are positive [9]. The delay in obtaining the result (even with the use of rapid methods) has led to the implementation of other diagnostic methods such as the dosage of adenosine deaminase (ADA). The dosage of this enzyme is usually elevated in tuberculous pleuritis but can also be high in other infectious diseases, rheumatoid arthritis, and even some malignancy [10]. Moreover, this assay is not available in all centers in developing countries. There are also other diagnostic tests available, such as Interferon and Polymerase Chain Reaction (PCR), the latter based on the amplification of mycobacterial DNA fragments. The disadvantages include the high cost, the risk of contamination, and the need of trained personnel.

In contrast, the diagnosis of malignant effusion depends on the volume of the sample submitted for the analysis and its cellularity. Usually, it requires the help of invasive procedures [11]. No single pleural fluid tumor marker is accurate enough for routine use in the diagnostic evaluation of pleural effusion [12]. Other predictive rules have been published for differentiating tuberculous pleurisy from malignant effusion [13,14,15,16]; however, these include the determination of ADA or other tests which are not widely available.

Our study has some limitations that must be acknowledged: we analyzed a single-center population and we do not have data on ADA determination in pleural fluid since this test is not always available in our setting. More studies are necessary to confirm these results.

## 5. Conclusions

The score we propose had a high sensitivity and specificity for the diagnosis of tuberculous pleural effusion. We think that it could provide a useful tool in the diagnosis of pleural effusion by means of a set of widely available variables.

## Figures and Tables

**Table 1:** Baseline characteristics. Data are media (SD) or median (IQR).

	Tuberculosis	Neoplasia	*p*-Value
	*n* = 47	*n* = 35	
Age (years)	37.6 (±18)	61.9 (±14)	<0.0001
Gender (Male/Female)	30/17	20/15	0.62
Lactic Acid, mmol·L^−1^	4.9 (3.52–6.00)	2.4 (1.82–3.72)	<0.0001
pH	7.30 (7.30–7.40)	7.40 (7.35–7.40)	0.004
Glucose, g·L^−1^	0.7 (0.60–0.81)	1.04 (0.92–1.35)	<0.0001
Proteins, g·L^−1^	5.1 (4.5–5.7)	3.95 (2.3–4.8)	<0.0001
LDH, mmol·L^−1^	868 (533–1289)	355 (272–585)	<0.0001
White cell count	1600 (897–3067)	650 (350–1770)	0.0052

**Table 2:** Multidimensional score.

Variables	Points
**Lactic Acid**	
<2.8 mmol·L^−1^	1
2.8–4.8 mmol·L^−1^	2
>4.8 mmol·L^−1^	3
**Glucose**	
>1.2 g·L^−1^	1
0.75–1.2 g·L^−1^	2
<0.75 g·L^−1^	3
**Age**	
>60 years	1
37–60 years	2
<37 years	3
**Proteins**	
<3.5 g·L^−1^	1
3.5–5 g·L^−1^	2
>5 g·L^−1^	3
